# Predictors of Adverse Outcomes During 30-Day Follow-Up in Patients Aged 75 Years and Older with Acute Coronary Syndrome

**DOI:** 10.3390/medicina62050923

**Published:** 2026-05-09

**Authors:** Arzu Arifovna Gurbanova, Kristina Gennadievna Pereverzeva, Valeria Vasilievna Trofimova, Valeria Valerievna Shitova, Sergey Stepanovich Yakushin

**Affiliations:** Department of Hospital Therapy with a Course of Medical and Social Expertise, Ryazan State Medical University, Ryazan 390000, Russia; pereverzevakg@gmail.com (K.G.P.); valeriatrofimova62@gmail.com (V.V.T.); lerochka.shitovaaa@mail.ru (V.V.S.); ssyakushin@yandex.ru (S.S.Y.)

**Keywords:** frailty, infarct-related coronary artery occlusion, IRCAO, acute coronary syndrome, myocardial infarction, total coronary occlusion, aged 75 and over, percutaneous coronary intervention, mortality, prognosis

## Abstract

***Background and Objectives*****:** The prognostic value of frailty and infarct-related coronary artery occlusion (IRCAO) in very old patients with acute coronary syndrome (ACS) remains insufficiently studied. This study aimed to identify risk factors for 30-day mortality in patients aged ≥75 years with myocardial infarction, depending on the presence of IRCAO and frailty. ***Materials and Methods*****:** A total of 360 patients (median age 80 [77; 84] years, 61.7% women) with ACS or acute myocardial infarction (AMI) were enrolled. Frailty was screened using the “Age is Not a Barrier” questionnaire in 228 patients. IRCAO was defined as TIMI 0–1 flow, absence of developed collateral circulation, and concordance between electrocardiogram changes and the affected coronary artery. The primary endpoint was all-cause mortality at 30 days. Kaplan–Meier survival analysis, Cox regression, and propensity score matching (PSM) were used. ***Results*****:** Frailty was present in 21.9% (50/228) of screened patients and was associated with significantly lower 30-day survival (88.0% vs. 97.2%, log-rank *p* = 0.015). Frailty increased the risk of death by 4.5-fold (HR = 4.474; 95% CI: 1.365–14.663; *p* = 0.013). IRCAO was detected in 20.0% (72/360) and was a significant predictor in the overall cohort (HR = 1.792; 95% CI: 1.031–3.112; *p* = 0.038), but its significance disappeared in the frailty-screened subgroup. Age remained an independent predictor only in the overall cohort. The unadjusted association between percutaneous coronary intervention and higher mortality (HR = 2.37) was explained by confounding by indication; after PSM, no difference in mortality persisted (*p* = 1.000). ***Conclusions*****:** Frailty is a powerful independent predictor of 30-day mortality in very old patients with AMI, outweighing chronological age and IRCAO. The prognostic value of IRCAO is mediated by frailty. The apparent harmful effect of percutaneous coronary intervention is explained by symptom severity rather than the procedure itself. Routine frailty assessment should be integrated into risk stratification for elderly ACS patients.

## 1. Introduction

Coronary artery disease (CAD) is the leading cause of mortality, morbidity, and disability in most countries and regions, including the Russian Federation [1]. Among patients with CAD, acute coronary syndrome (ACS) poses a serious problem due to major adverse cardiovascular events during hospitalization and post-discharge follow-up. In-hospital mortality from ACS in the Russian Federation in 2022 was 5.6% [1]. In-hospital mortality among patients diagnosed with acute myocardial infarction (AMI) was 10.9% [1].

Primary percutaneous coronary intervention (PCI) is first-line treatment for ST-segment elevation myocardial infarction (STEMI) when performed within 12 h of symptom onset and 120 min of diagnosis. It reduces mortality compared with other reperfusion strategies [1]. For the management of non-ST-segment elevation ACS (NSTE-ACS), several invasive treatment algorithms have been developed over the years. According to current guidelines, the first step is to determine the risk for each patient in the acute phase, followed by the choice of an immediate, early, or selective invasive strategy depending on the risk class [2]. In elderly and very old patients with STEMI or NSTE-ACS, the same diagnostic and treatment strategies are recommended as in younger patients—to improve their clinical status and prognosis [1,2]. However, there is currently no substantial body of evidence showing that a routine invasive strategy leads to better outcomes in very old patients than a conservative approach, particularly in terms of reducing mortality. The SENIOR-RITA trial (n = 1518, aged ≥ 75 years with non-ST-segment elevation myocardial infarction (NSTEMI)) randomized patients to invasive vs. conservative strategy. Frail and cognitively impaired patients were included. The primary endpoint (cardiovascular death/myocardial infarction (MI)) and mortality did not differ between strategies, but MI and revascularization risk were significantly reduced [3].

Patients with STEMI have higher short-term mortality compared to those with NSTEMI [4,5]. This trend changes during 1–2 years of follow-up, when mortality rates become comparable [6]. This is likely explained by differences in baseline patient characteristics, including older age and a higher prevalence of comorbidities in the NSTEMI group, as well as the fact that many NSTEMI patients do not receive adequate treatment due to incorrect risk stratification. One reason may be electrocardiogram (ECG)–coronary artery (CA) discordance. STEMI typically follows acute complete or near-complete CA occlusion, but complete infarct-related artery occlusion also occurs in NSTEMI [7,8]. ECG has limited sensitivity for posterior or lateral wall ischemia (often due to left circumflex artery occlusion), explaining why some complete occlusions present as NSTEMI [7]. Large retrospective studies and meta-analyses of NSTEMI patients have shown that approximately 25–30% have complete artery occlusion, and 34% have Thrombolysis in Myocardial Infarction (TIMI) flow grade 0 to 1 [9,10]. Notably, among NSTEMI patients with complete CA occlusion, the mortality rate is nearly twice as high as in NSTEMI patients without CA occlusion, despite the fact that they are younger and have fewer comorbidities [10].

Although age, as a crucial prognostic marker, is included in most ACS risk assessment tools, including the TIMI and Global Registry of Acute Coronary Events (GRACE) scores, and determines a high risk of adverse outcomes [8], this risk is often overlooked due to the presence of cognitive impairment and frailty in the patient [11]. Among elderly and very old patients, in-hospital mortality from MI, without distinction between STEMI and NSTEMI, is 27.8% [12], whereas in-hospital mortality from MI in the general population is 10.9% [2]. Such data are likely related to the presence of frailty syndrome in these patient categories. Frailty independently predicts all-cause mortality in ACS patients ≥ 75 years. Man et al. reported a frailty prevalence of 19.7–48.5% in elderly ACS patients [13]. Frail patients had a 5.49-fold higher risk of in-hospital death, a 3.56-fold higher risk of short-term mortality, and a 2.39-fold higher risk of long-term mortality [13].

Aim—to identify risk factors for death during 30-day follow-up in patients aged 75 years and older with MI, depending on the presence of infarct-related coronary artery occlusion (IRCAO) and frailty.

## 2. Materials and Methods

### 2.1. Study Design and Population

This is a prospective and single-center study. Patient enrollment was conducted at a cardiology hospital from June 2024 to June 2025. Inclusion criteria were: age ≥ 75 years, diagnosis of ACS or AMI (onset ≤ 7 days) upon admission, signed voluntary informed consent, and coronary angiography performed during the index hospitalization. The only exclusion criterion was the patient’s withdrawal of voluntary informed consent to participate in the observational program.

Upon admission, patients underwent screening for frailty using the “Age is Not a Barrier” questionnaire, which has been officially validated in the Russian population [14]. It consists of 7 questions covering weight loss, sensory deficits, falls, symptoms of depression and cognitive impairment, urinary incontinence, and reduced mobility. Due to various circumstances (severe patient condition, patient refusal to undergo additional tests), frailty screening was performed in 228 patients. A score of ≥5 points confirmed the presence of frailty (sensitivity of 85.7–93.3%, showing good agreement with the Fried phenotype and Rockwood index). Frailty assessment was not performed in patients with an extremely serious condition (hemodynamic instability, respiratory failure, impaired consciousness) or if the patient/relatives refused additional tests.

During the study, a group of patients with IRCAO was identified. Inclusion criteria for this group were: the presence of TIMI 0 or 1 flow on coronary angiography, absence of antegrade filling of the distal bed, absence of collateral blood flow, and consistency of ECG changes with the CA lesion on angiography. Systematic assessment of inter-rater variability was not performed.

All decisions regarding the performance of PCI, the choice of revascularisation method, or the decision to withhold intervention were made by the treating medical team (interventional cardiologists, cardiac surgeons, anaesthesiologists) in strict accordance with current clinical guidelines [15,16]. The research team played an observational role and did not interfere with the treatment process.

Criteria for performing PCI were based on the following principles.

For primary PCI in patients with STEMI: (1) mandatory intervention on the infarct-related artery (resolution of acute thrombotic occlusion of a major coronary artery branch) to reduce the risk of death. Evidence for this is derived from meta-analyses comparing PCI on the infarct-related artery with thrombolytic therapy. (2) To reduce the overall risk of ischaemic events (death, recurrent myocardial infarction, repeat revascularisation), PCI on non-infarct-related arteries was recommended in haemodynamically stable patients with STEMI and multivessel disease, preferably as a staged procedure (during the index hospitalisation or within the following weeks, but not earlier than 72 h after the primary intervention). When the SYNTAX (Synergy between PCI with TAXus and Cardiac Surgery) score was >23, an immediate or telemedicine consultation with a cardiac surgeon was obtained to consider CA bypass grafting (CABG).

For PCI in patients with NSTEMI: (1) in patients with single-vessel disease, PCI on the symptom-related stenosis (occlusion) was performed immediately after coronary angiography to reduce the risk of recurrent MI. (2) In patients with multivessel disease, the choice of revascularisation method (PCI vs. CABG) required a dedicated discussion taking into account the patient’s clinical condition, preferences, the extent and characteristics of coronary atherosclerosis, comorbidities, the feasibility of long-term dual antiplatelet therapy, and the need for long-term anticoagulation. The use of the SYNTAX score and the same principles as for patients with stable CAD was recommended. The decision regarding complete or incomplete, simultaneous or staged revascularisation was made on an individual basis.

Definition of successful PCI. In our study, a patient was considered to have undergone PCI only if the procedure was successful (achievement of TIMI 2–3 flow in the target artery). Patients in whom the procedure was not completed (including the 4 patients who died on the operating table before the start of PCI) were not included in the PCI group.

The overall patient group was divided into 2 subgroups depending on the outcome at 30 days from the time of hospitalization.

### 2.2. Variables

The variables analyzed were: age, gender, change in the ST segment of the ECG upon admission, diagnosis upon admission, diagnosis upon discharge, outcome of hospitalization, presence or absence of IRCAO, PCI in the general group and for occlusion of infarct-related CA, score on the scale “Age is not a Barrier”, history of CAD, MI, hypertension, chronic heart failure (CHF), atrial fibrillation, complete left bundle branch block, cerebrovascular disease, acute cerebrovascular accident, type 2 diabetes mellitus, the presence of zones of hypo- and akinesia of the left ventricle according to echocardiography, the level of left ventricle ejection fraction (LVEF), the level of highly sensitive troponin I upon admission, the maximum level of creatine phosphokinase (CK) and its MV fraction (CK-MV), the level of total cholesterol, low-density lipoproteins, high-density lipoproteins, triglycerides, aspartate aminotransferase, alanine aminotransferase, serum creatinine, urea, uric acid, total protein, potassium and sodium, venous blood glucose, erythrocytes, hemoglobin, leukocytes, platelets, erythrocyte sedimentation rate, and glomerular filtration rate according to the CKD-EPI (Chronic Kidney Disease Epidemiology Collaboration) formula.

To assess the short-term prognosis, a binary variable, “status at 30 days”, was introduced, which took a value of 1 for patients who remained alive 30 days after hospitalization, and 0 for those who died during this period. The follow-up period was calculated from the time of admission to the hospital.

### 2.3. Outcome Variable

The primary endpoint was all-cause 30-day mortality in patients aged ≥75 years with ACS.

### 2.4. Statistical Analysis

Statistical analysis was performed using StatTech v. 4.10.3 (Stattech LLC, Moscow, Russia) and SPSS Statistics 26 (IBM Corp., Armonk, NY, USA).

Quantitative variables were assessed for conformity to a normal distribution using the Shapiro–Wilk test (for sample sizes less than 50) or the Kolmogorov–Smirnov test (for sample sizes greater than 50).

Quantitative variables with a sample distribution conforming to a normal distribution were described using arithmetic means (M) and standard deviations (SD). The 95% confidence intervals (95% CI) were reported as a measure of representativeness for the mean values.

In cases where the distribution was not normal, quantitative data were described using the median (Me) and lower and upper quartiles [Q1; Q3].

Categorical data were described using absolute values and percentages. The 95% confidence intervals for percentages were calculated using the Clopper–Pearson method.

Comparison of two groups regarding a quantitative variable with a normal distribution in each group was performed using Student’s *t*-test under the assumption of equal variances, and Welch’s *t*-test in cases of unequal variances.

Comparison of two groups regarding a quantitative variable with a non-normal distribution was performed using the Mann–Whitney U test.

Comparison of percentages in the analysis of four-fold contingency tables was performed using Pearson’s chi-squared test (for expected frequencies greater than 10) or Fisher’s exact test (for expected frequencies less than 10).

In cases of zero observations in contingency table cells, the odds ratio was calculated using the Haldane–Anscombe correction.

Comparison of percentages in the analysis of multi-field contingency tables was performed using Pearson’s chi-squared test.

All variables that demonstrated a statistically significant association with the outcome in the univariate analysis (*p* < 0.05) were included in the multivariate model. In total, 6 variables were entered; with 60 deaths observed, the events per variable was 60/6 = 10, meeting the minimum recommended threshold. Variables were entered using the forced entry method (Enter). Before building the model, multicollinearity was assessed using the variance inflation factor; the variance inflation factor values for all analysed predictors were below 2, indicating no significant collinearity. The variable IRCAO was not included in the model due to its high correlation with the performance of PCI (Pearson correlation coefficient r = 0.93).

Assessment of patient survival function was performed using the Kaplan–Meier method. The survival function estimate is represented as a descending stepwise curve, with survival function values between observation points considered constant.

Patient survival analysis was performed using Cox regression, which involves predicting the instantaneous risk of an event for a given subject at a specific point in time (hazard) and assessing the impact of predefined independent variables (predictors) on this risk. Hazard ratios with 95% confidence intervals (HR; 95% CI) were calculated, and the statistical significance of each predictor’s effect was assessed.

Given that frailty assessment was not performed in all patients (n = 228 out of 360), to analyze the impact of frailty and to ensure valid comparisons in multivariate models, further statistical analysis including the frailty variable was conducted on this patient sample (n = 228).

To reduce the effect of systematic differences between groups of patients who underwent PCI and those who did not, a pseudo-randomization method based on propensity score matching (PSM) was used. Propensity scores were calculated for each patient using multivariate logistic regression, where the dependent variable was PCI performance and the independent variables were characteristics potentially influencing selection into the groups: age, ECG ST-segment changes upon admission, presence or absence of IRCAO, LVEF level, high-sensitivity troponin I level upon admission, maximum CK level, venous blood glucose level, and leukocyte count. Patient matching was performed using the nearest neighbor matching algorithm without replacement at a 1:1 ratio with a caliper set to 0.01 of the standard deviation of the logit of the propensity score. Matching quality was assessed by analyzing the standardized mean difference (SMD) for all covariates included in the model. SMD values of less than 0.1 after the matching procedure were considered evidence of successful sample balancing.

Comparison of outcomes between the formed groups was performed using paired Student’s *t*-test.

Differences were considered statistically significant at *p* < 0.05. Variables that showed an association with the outcome at *p* < 0.05 in the univariate analysis were used to build the multivariate models.

### 2.5. Ethical Considerations

The study was conducted in accordance with the Declaration of Helsinki and approved by the Local Ethics Committee of Ryazan State Medical University (protocol code No. 9, date of approval: 11 March 2024). Informed consent was obtained from all subjects involved in the study.

## 3. Results

### 3.1. Baseline Characteristics

A total of 360 patients were enrolled, with a median age of 80 [77; 84] years, of whom 222 (61.7%) were women.

Among the included patients, IRCAO was detected in 20.0% (n = 72) of cases. Of these, 26.4% (n = 19) had no ST-segment elevation on ECG upon admission.

As shown in Table 1, a comparative analysis of clinical, demographic, and anamnestic characteristics between the groups of survivors and those who died within 30 days revealed no statistically significant differences, except for differences in age and the presence of chronic heart failure.

As shown in Table 2, a comparative analysis of laboratory and instrumental characteristics between the groups of survivors and those who died within 30 days revealed statistically significant differences: non-survivors had higher levels of myocardial necrosis biomarkers, creatinine, alanine aminotransferase, aspartate aminotransferase, urea, uric acid, leukocytes, and blood glucose; lower levels of total serum protein; lower estimated glomerular filtration rate; lower LVEF on the first day; and a higher frequency of IRCAO and zones of hypo- and akinesis of the myocardium on the first day.

Frailty screening was performed in 228 out of 360 patients (63.3%). The main reasons for the absence of screening were severe condition at admission (n = 36) and patient refusal to undergo additional tests (n = 96). Patients without frailty assessment had higher creatinine, urea, and prevalence of cerebrovascular disease, and lower estimated glomerular filtration rate. In-hospital and 30-day mortality were significantly higher in the non-screened group (36.4% and 37.1%) than in the screened group (3.1% and 4.8%; *p* < 0.001 for both) (Table 3). For all other clinical, laboratory and instrumental parameters analyzed in our study, no statistically significant differences were found (*p* ≥ 0.05 for all).

### 3.2. Outcome Variable

In-hospital mortality in the overall group of patients aged 75 years and older was 15.3% (n = 55). Mortality within one month was 16.7% (n = 60).

### 3.3. Univariable and Multivariable Analysis

Frailty was identified in 21.9% (n = 50) of the 228 patients who underwent screening. Of these, 28% (n = 14) had IRCAO, and 92.9% (n = 13) of them underwent PCI. Overall, 80% (n = 40) of frail patients underwent PCI during hospitalization. Frailty was statistically significantly more common in women (82.0% (n = 41)) than in men (18.0% (n = 9)), *p* < 0.001.

Univariate analysis showed that the presence of IRCAO in the overall group (n = 360) was significantly associated with 30-day mortality. The odds of death in the IRCAO group were 2.0 times higher (95% CI: 1.044–3.65; *p* = 0.034).

However, in the sample of patients who underwent frailty screening (n = 228), the presence of IRCAO was not significantly associated with mortality (72.7% (n = 8) of deaths in patients without IRCAO vs. 27.3% (n = 3) of deaths in patients with IRCAO, *p* = 0.448), and age also did not differ between outcome groups (median age of survivors 79.0 [76.0; 84.0] (n = 216) vs. median of deceased 84.0 [77.5; 87.5] (n = 11), *p* = 0.253).

In the sample of patients who underwent frailty screening (n = 228), multivariate survival analysis using Cox regression was performed including age, IRCAO, and frailty. However, the effect of age and IRCAO in this model did not reach statistical significance (*p* > 0.05).

### 3.4. Survival

In the analysis of the sample of patients who underwent frailty screening (n = 228), frail patients had a statistically significantly higher mortality rate compared to patients without frailty (12.0% (n = 6) vs. 2.8% (n = 5), *p* = 0.016).

**Figure 1:**
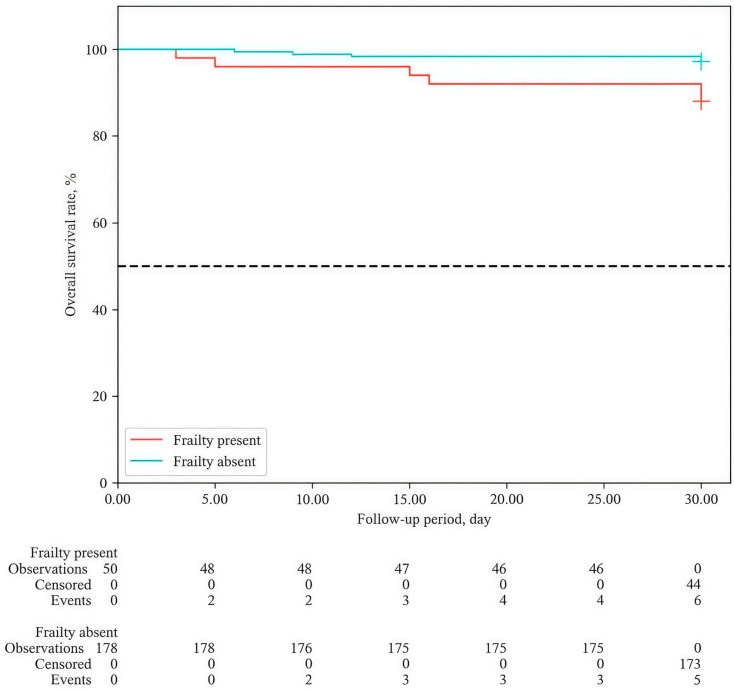
Overall survival (Kaplan–Meier) curves depending on the presence of frailty during the 30-day follow-up period.

As shown in Figure 1, the presence of frailty was significantly associated with lower overall survival (log-rank test, *p* = 0.015). Frail patients demonstrated substantially worse survival compared to patients without frailty at all time points of follow-up. By day 30, cumulative survival in the frail group was 88.0% (95% CI: 75.2–94.4) compared to 97.2% (95% CI: 93.4–98.8) in the non-frail group. The presence of frailty was associated with a 4.5-fold higher risk of death (HR = 4.474 (95% CI: 1.365–14.663; *p* = 0.013)). Neither the median nor the 25th/75th percentiles of survival were reached in either group within 30 days.

**Figure 2:**
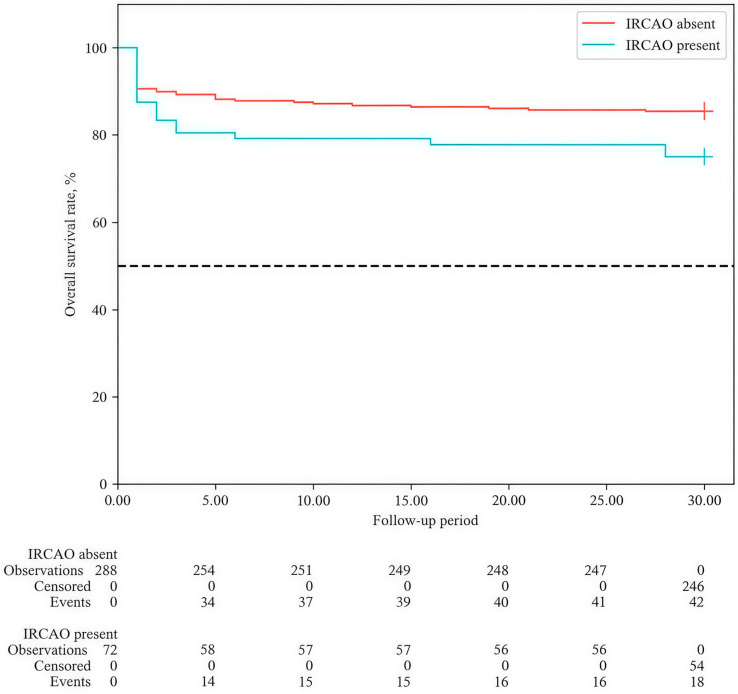
Overall survival (Kaplan–Meier) curves depending on the presence of infarct-related coronary artery occlusion (IRCAO) during the 30-day follow-up period.

Survival analysis depending on the presence of IRCAO also revealed statistically significant differences (HR = 1.792 (95% CI: 1.031–3.112; *p* = 0.038)) (Figure 2). Patients with IRCAO had statistically significantly worse survival at all analyzed time points. Neither the median nor quartiles of survival were reached within 30 days in either group, indicating a relatively small total number of deaths during the observation period. By day 30 of follow-up, cumulative survival in the group without IRCAO was 85.4% (95% CI: 80.8–89.0%). In the IRCAO group, survival was statistically significantly lower—75.0% (95% CI: 63.3–83.4%).

**Figure 3:**
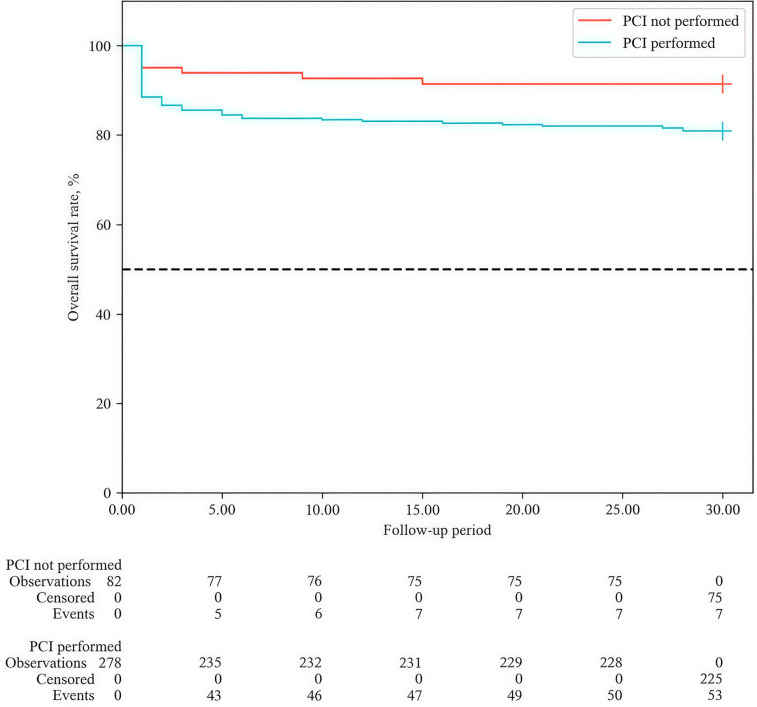
Overall survival (Kaplan–Meier) curves depending on the performance of percutaneous coronary intervention (PCI) during the 30-day follow-up period.

The frequency of PCI in the overall group was 77.2% (n = 278). Unadjusted analysis showed higher 30-day mortality in the PCI group (HR = 2.37; 95% CI: 1.078–5.216; *p* = 0.032). Kaplan–Meier curves showed lower cumulative survival in the PCI group at all time points (Figure 3). By day 30, survival was 91.5% (95% CI: 82.9–95.8%) in the non-PCI group vs. 80.9% (95% CI: 75.8–85.1%) in the PCI group (*p* = 0.017).

As shown in Table 2, the frequency of PCI performed on the IRCAO among deceased patients was statistically higher, *p* = 0.007. However, correlation analysis showed a Pearson correlation coefficient of 0.93 between IRCAO and PCI on the IRCAO, *p* < 0.001, indicating a strong positive correlation. IRCAO was excluded from the analysis.

In the overall patient group (n = 360), multivariate survival analysis using Cox regression was performed including age and PCI on the IRCAO. In the multivariable Cox model that included age and PCI on the IRCAO, PCI on the IRCAO was associated with an adjusted HR of 2.05 (95% CI: 1.182–3.573; *p* = 0.011), and age was associated with an adjusted HR of 1.10 (adjusted HR = 1.10; 95% CI: 1.053–1.159; *p* < 0.001).

### 3.5. Propensity Score Matching

To mitigate differences in baseline characteristics between groups of patients who underwent PCI and those who did not, the PSM method was applied. The groups were balanced according to the following parameters: age, ECG ST-segment changes upon admission, presence or absence of IRCAO, LVEF value, high-sensitivity troponin I level upon admission, maximum CK level, venous blood glucose level, and leukocyte count. A total of 70 pairs were obtained.

The original sample included 278 patients who underwent PCI and 82 patients who did not undergo PCI. As a result of matching by propensity score using the nearest neighbor algorithm (1:1, caliper 0.01), 70 comparable pairs were formed. For 208 patients in the main group, no suitable pairs were found in the control group within the specified caliper, and therefore they were excluded from further analysis.

Assessment of covariate balance after matching is presented in Table 3. The SMD for all features included in the model did not exceed 0.1, confirming the high quality of group matching (Table 4).

After matching, no difference in mortality was found (*p* = 1.000). However, 75% (208/278) of PCI patients were excluded from the matched analysis, limiting generalisability. Residual confounding cannot be fully excluded.

### 3.6. Risk on the GRACE Scale

When comparing patients according to risk level by the GRACE scale (version 1.0), statistically significant differences were identified regarding the frequency of PCI and status at 30 days (Table 5). The frequency of PCI performance differed significantly between risk groups (*p* < 0.001). The highest proportion of patients undergoing the intervention was observed in the high-risk group (81.8%), whereas in the low- and moderate-risk groups this rate was 60.0% and 51.1%, respectively. In pairwise comparison between the high- and moderate-risk groups, the differences were also statistically significant (*p* ≤ 0.001). Mortality at 30 days differed significantly between groups (*p* = 0.014). The highest proportion of deaths was recorded in the high-risk group (19.1%), while in the moderate-risk group 4.3% of patients died, and in the low-risk group all patients survived (100.0%). When comparing the high- and moderate-risk groups, the differences also reached statistical significance (*p* = 0.035).

Among high-risk patients according to the GRACE score (≥141 points), 55 individuals were not revascularised for the following reasons: 4 patients died on the operating table before the start of PCI, 14 patients required CABG (two of them had a history of CABG), 37 patients had no angiographic criteria for PCI (two of them had a history of CABG). Of these 55 patients, four received thrombolytic therapy at the prehospital stage, after which three did not have angiographic criteria for PCI, and one required CABG.

## 4. Discussion

The results of this study demonstrate that frailty is a powerful independent predictor of 30-day mortality in patients ≥75 years after ACS, increasing the risk of death approximately 4.5-fold. This effect persists after adjustment for chronological age and the presence of IRCAO. Our findings are consistent with a meta-analysis by Man et al. (2019), who reported a 5.49-fold increase in in-hospital mortality and a 3.56-fold increase in short-term mortality in frail elderly patients with ACS [13]. The prevalence of frailty in our cohort (21.9%) also matches literature data [13]. Frailty is not merely a surrogate for age or severe coronary pathology; it provides independent prognostic information beyond these factors. It should be considered that frailty was not assessed in the most critically ill patients at presentation; therefore, the prognostic impact of frailty reported in this study may be underestimated, and our findings likely apply primarily to haemodynamically stable elderly patients with AMI.

As expected, age remained an independent risk factor in the overall cohort (n = 360). However, when we focused on the subgroup with frailty assessment (n = 228), the significance of age and IRCAO disappeared, and frailty emerged as the dominant predictor. This shift underscores that biological age and physiological reserve, captured by frailty, often outweigh chronological age or isolated anatomical findings [11,13].

**The PCI paradox and confounding by indication.** At first glance, the higher 30-day mortality in patients who underwent PCI (88.3% vs. 75.0%, *p* = 0.025; unadjusted HR = 2.37) seems contradictory. This finding, however, reflects clinical reality—patients with the most severe ACS (high GRACE score, IRCAO, elevated biomarkers) were more likely to be referred for invasive revascularisation (“confounding by indication”). In a multivariate Cox model including age and PCI on the IRCAO, PCI on the IRCAO remained associated with a higher risk (adjusted HR = 2.05), indicating that the intervention itself is not the cause but rather a marker of baseline severity. To test this hypothesis, PSM was performed (70 pairs, balanced for age, ST-segment changes, troponin, CK, glucose, leukocytes, LVEF, IRCAO). After matching, mortality no longer differed between PCI and non-PCI groups (*p* = 1.000). Importantly, PCI was not performed in 55 high-risk GRACE patients for objective reasons (death on the table, need for CABG, absence of angiographic criteria for PCI), not because of a default conservative strategy. Thus, the elevated risk observed in the unadjusted PCI group reflects patients’ baseline severity, not an adverse effect of the procedure.

**Sex and age distribution.** The predominance of women (61.7%) in our very elderly cohort (≥75 years) might appear to contradict the higher CAD prevalence in men. This is explained by demographic and hormonal factors. According to Rosstat (2024), women outnumber men in the Russian Federation (78.5 vs. 67.7 million); among individuals ≥ 85 years, women outnumber men by 3.3-fold [17]. International registries confirm similar trends. The FINAMI study [18] reported that 65% of CAD events in the 75–99 age group occurred in women, with rising MI incidence in women 85–99 years. Perl et al. [19] found that women comprised 40.8% of octogenarians with STEMI versus 31.9% of septuagenarians and 26.5% of younger patients. The French e-MUST registry [20] showed that women represented only 22% of all STEMI patients but >60% of those ≥ 90 years. After menopause, loss of oestrogen-mediated cardioprotection (reduced inflammation, decreased matrix metalloproteinase expression, improved endothelium-dependent vasodilation) equalises CAD risk between sexes [21,22,23,24,25,26,27,28]. Hence, the female predominance in our study reflects demographic realities and postmenopausal hormonal changes, not a contradiction.

**Chronic heart failure paradox.** In our study, a history of CHF was unexpectedly more common among survivors (13.0% vs. 3.4% in non-survivors, *p* = 0.041). This counter-intuitive finding likely results from the protective effect of baseline CHF therapy (angiotensin-converting enzyme inhibitors; angiotensin II receptor blockers; beta-adrenergic blocking agents; aldosterone antagonists; sodium-glucose co-transporter 2 inhibitors). Patients regularly receiving such medication may experience less myocardial damage during an acute event, improving short-term survival [29]. Other possible mechanisms include ischaemic preconditioning and survivorship bias. We note that only pre-existing CHF was analysed; the development of de novo CHF after AMI was not studied.

**Clinical implications.** The strong independent association of frailty with 30-day mortality suggests that routine frailty screening should be incorporated into risk stratification for elderly ACS patients. The lack of a true adverse effect of PCI after appropriate adjustment supports an invasive strategy when clinically indicated, even in the very old.

## 5. Study Limitations

Our study has several limitations that should be considered when interpreting the results.

**Data on prior medications.** We did not collect detailed information on medications taken before hospitalisation. Therefore, the observed association between a history of CHF and better survival remains speculative, as it may be explained by the protective effect of baseline CHF therapy (angiotensin-converting enzyme inhibitors; angiotensin II receptor blockers; beta-adrenergic blocking agents, etc.).

**Limited assessment of CHF.** Only a history of CHF (diagnosed before the acute event) was analysed; the development of de novo CHF after MI was not studied. Moreover, survivorship bias cannot be excluded—patients with CHF who survived to ≥75 years may represent a selected frailer but still resilient subgroup.

**Single-centre design and sample size.** The single-centre design limits generalisability to other healthcare systems and populations. The relatively small number of events (60 deaths) affected the precision of estimates, especially in subgroup analyses.

**Incomplete frailty screening.** Frailty assessment was performed in only 63% of patients (228/360) due to severe condition at admission (n = 36) or patient refusal (n = 96). Patients without frailty screening were significantly older, had worse renal function, lower LVEF, more frequent CHF, and markedly higher 30-day mortality (37.1% vs. 4.8%). Consequently, the missing data are not random (MNAR). This selection bias should be considered when interpreting frailty-related results; however, our main multivariate models that did not include frailty accounted for key severity factors, partly reducing the risk.

**Competing risks.** Competing risks (e.g., death from cancer, infections, or other non-cardiovascular causes) were not taken into account in the survival analysis. This may overestimate the incidence of cardiovascular death, particularly in very old patients where non-cardiovascular mortality is substantial.

**Limitations related to PCI and revascularisation.**

• The study is observational, not randomised. All revascularisation decisions were made by the treating medical team, leading to potential confounding by indication—more severe patients were more likely to undergo PCI. Despite multivariable adjustment and PSM, residual confounding cannot be completely eliminated.
• Patients who died on the operating table before the start of PCI were assigned to the non-PCI group, which may have biased the comparison.
• No standardised protocol existed for patients with multivessel disease; the choice between PCI and CABG depended on clinical judgment, SYNTAX score, LVEF, and patient preferences.
• In the 37 patients who did not undergo PCI, significant CAD was ruled out solely by coronary angiography, without routine use of intracoronary imaging (optical coherence tomography or intravascular ultrasound) due to lack of equipment, potentially missing non-stenotic lesions.

**Definition of IRCAO and inter-rater variability.** Complete occlusion of the infarct-related artery was defined using TIMI 0–1 flow, absence of collateral filling, and ECG correlation. Formal inter-rater reliability assessment was not performed, which could introduce some misclassification bias.

## 6. Conclusions

1. Frailty is a powerful independent predictor of 30-day mortality in screened patients ≥75 years with AMI (HR = 4.47). Its prognostic strength outweighs chronological age and anatomical severity of coronary artery involvement.

2. These data support mandatory frailty assessment in risk stratification for ACS patients ≥75 years. This will facilitate balanced clinical decision-making, including the appropriateness and potential benefit of an invasive strategy.

## Figures and Tables

**Table 1:** Clinical, demographic, and anamnestic parameters of survivors and non-survivors.

Parameters	Total Group n = 360	Status at 30 Days		*p*
		Non-Survivors n = 60	Survivors n = 300	
**Age, years, Me [IQR]**	80 [77.0; 84.0]	83.0 [78.0; 85.3]	79.0 [76.0; 84.0]	<0.001 *
**Female sex, n (%)**	222 (61.7%)	41 (68.3%)	181 (60.3%)	0.245
**History of coronary artery disease, n (%)**	219 (60.8%)	31 (51.7%)	188 (62.7%)	0.111
**History of myocardial infarction, n (%)**	145 (40.3%)	22 (36.7%)	123 (41.0%)	0.532
**History of hypertension, n (%)**	352 (97.8%)	59 (98.3%)	293 (97.7%)	1.000
**History of chronic heart failure, n (%)**	41 (11.4%)	2 (3.4%)	39 (13.0%)	0.041 *
**History of atrial fibrillation, n (%)**	95 (26.4%)	12 (20.0%)	83 (27.7%)	0.219
**History of complete left bundle branch block, n (%)**	8 (2.2%)	3 (5.1%)	5 (1.7%)	0.243
**History of cerebrovascular disease, n (%)**	145 (40.3%)	30 (50.0%)	115 (38.3%)	0.093
**History of acute stroke, n (%)**	48 (13.3%)	13 (21.7%)	35 (11.7%)	0.058
**Type 2 diabetes mellitus, n (%)**	118 (32.8%)	21 (35.0%)	97 (32.3%)	0.688

*—statistically significant differences (*p* < 0.05); bold font in the table footer is used for better visual guidance.

**Table 2:** Laboratory and instrumental parameters of survivors and non-survivors.

Parameters	Total Group n = 360	Status at 30 Days		*p*
		Non-Survivors n = 60	Survivors n = 300	
**Admission diagnosis NSTE-ACS, n (%)**	196 (54.4%)	23 (38.3%)	173 (57.7%)	0.006 *
**Admission diagnosis STE-ACS, n (%)**	164 (45.6%)	37 (61.7%)	127 (42.3%)	
**Discharge diagnosis NSTEMI, n (%)**	152 (42.2%)	25 (42.4%)	127 (42.3%)	0.923
**Discharge diagnosis STEMI, n (%)**	150 (41.7%)	34 (56.7%)	116 (38.7%)	0.008 *
**Discharge diagnosis unstable angina stabilized into stable angina, n (%)**	55 (15.3%)	1 (1.7%)	54 (18.0%)	0.007 *
**Alternative discharge diagnosis, n (%)**	3 (0.8%)	0 (0.0%)	3 (1.0%)	0.579
**Troponin I on admission, ng/L, Me [IQR]**	1034.0 [62.0; 8228.0]	3045.5 [686.5; 19,342.8]	802.0 [36.6; 6672.0]	0.002 *
**Creatine kinase max., U/L, Me [IQR]**	469.3 [179.6; 1127.8]	864.0 [230.4; 2117.1]	457.2 [179.6; 1023.5]	0.023 *
**Creatine kinase MB fraction max., U/L, Me [IQR]**	59.0 [26.3; 144.0]	100.1 [41.7; 292.3]	54.5 [25.7; 129.5]	0.001 *
**Creatinine max., µmol/L, Me [IQR]**	111.4 [93.0; 150.0]	182.8 [113.9; 269.7]	108.80 [92.07; 139.30]	<0.001 *
**GFR by CKD-EPI formula, mL/min/1.73 m^2^, M ± SD**	45.9 ± 19.0 [43.9; 47.9]	30.0 [15.0; 39.0]	47.8 [36.5; 61.0]	<0.001 *
**Aspartate aminotransferase max., U/L, Me [IQR]**	46.6 [26.2; 109.9]	154.8 [50.9; 307.6]	43.6 [25.2; 84.3]	<0.001 *
**Alanine aminotransferase max., U/L, Me [IQR]**	28.3 [17.8; 43.9]	48.3 [32.0; 117.3]	27.3 [17.7; 41.1]	<0.001 *
**Glucose max., mmol/L, Me [IQR]**	8.1 [6.7; 10.8]	10.7 [8.1; 14.8]	7.9 [6.6; 10.1]	<0.001 *
**Urea max., mmol/L, Me [IQR]**	8.7 [6.9; 13.2]	16.2 [10.6; 21.4]	8.5 [6.7; 12.1]	<0.001 *
**Uric acid max., Me [IQR]**	398.3 [317.6; 477.5]	483.3 [372.0; 622.7]	390.8 [311.3; 465.7]	<0.001 *
**Total protein max., g/L, M (SD)**	69.2 ± 6.8 [68.4; 69.9]	66.9 (7.8)	69.4 (6.6)	0.039 *
**Total cholesterol max., mmol/L, Me [IQR]**	4.9 [4.0; 5.7]	5.2 [4.1; 6.3]	4.8 [4.0; 5.7]	0.226
**Low-density lipoproteins max., mmol/L, Me [IQR]**	2.9 [2.2; 3.8]	3.2 [2.2; 4.3]	2.8 [2.1; 3.7]	0.190
**High-density lipoproteins max., mmol/L, Me [IQR]**	1.3 [1.1; 1.5]	1.1 [1.1; 1.5]	1.3 [1.1; 1.5]	0.363
**Triglycerides max., mmol/L, Me [IQR]**	1.3 [0.9; 1.7]	1.4 [1.0; 1.7]	1.3 [0.9; 1.7]	0.625
**Potassium, mmol/L, M ± SD**	4.3 ± 0.7 [4.3; 4.4]	4.4 (0.9)	4.3 (0.6)	0.887
**Sodium, mmol/L, Me [IQR]**	142.0 [140.0; 144.0]	141.4 [138.0; 144.0]	142.0 [140.0; 144.0]	0.140
**Erythrocytes min., ×** **10^12^/L, Me [IQR]**	4.1 [3.8; 4.5]	4.0 [3.6; 4.4]	4.1 [3.8; 4.5]	0.309
**Hemoglobin min., g/L, M ± SD**	119.9 ± 18.5 [117.9; 121.8]	115.6 (20.6)	120.5 (18.1)	0.109
**Erythrocyte sedimentation rate, mm/h, Me [IQR]**	36.0 [26.0; 49.0]	32.5 [17.0; 45.5]	36.0 [26.5; 49.5]	0.214
Leukocytes max., *10^9^/L, Me [IQR]	10.7 [8.0; 13.5]	14.8 [10.8; 19.4]	10.3 [7.8; 12.6]	<0.001 *
Platelets max., *10^9^/L, Me [IQR]	206.5 [172.0; 241.8]	228.0 [179.5; 270.5]	205.0 [172.0; 241.0]	0.213
**Zones of left ventricular hypo- and akinesis, n (%)**	280 (83.8%)	35 (100.0%)	245 (81.9%)	0.003 *
**Left ventricular ejection fraction on day 1, %, Me [IQR]**	48.0 [44.0; 56.0]	44.0 [37.0; 48.0]	50.0 [44.3; 56.0]	<0.001 *
**IRCAO, n (%)**	72 (20.0%)	18 (30.0%)	54 (18.0%)	0.034 *
**PCI, n (%)**	278 (77.2%)	53 (88.3%)	225 (75.0%)	0.025 *
**PCI on IRCAO, n (%)**	64 (88.9%)	18 (30.0%)	46 (15.3%)	0.007 *

Note: NSTE-ACS—non-ST-segment elevation acute coronary syndrome; STE-ACS—ST-segment elevation acute coronary syndrome; NSTEMI—non-ST-segment elevation myocardial infarction; STEMI—ST-segment elevation myocardial infarction; GFR by CKD-EPI—glomerular filtration rate by Chronic Kidney Disease Epidemiology Collaboration formula; IRCAO—infarct-related coronary artery occlusion; PCI—percutaneous coronary intervention. *—statistically significant differences (*p* < 0.05); bold font in the table footer is used for better visual guidance.

**Table 3:** Comparative characteristics of patients with and without frailty screening.

Parameters	Total Group n = 360	Frailty Screening		*p*
		Yes n = 228	No n = 132	
**Creatinine max., µmol/L, Me [IQR]**	111.4 [93.0; 150.0]	109.5 [92.4; 139.3]	121.4 [96.2; 165.3]	0.046 *
**GFR by CKD-EPI formula, mL/min/1.73 m^2^, M (SD)**	45.9 ± 19.0 [43.9; 47.9]	47.4 (18.6)	43.0 (19.6)	0.048 *
**Urea max., mmol/L, Me [IQR]**	8.7 [6.9; 13.2]	8.5 [6.7; 12.4]	9.5 [7.3; 14.2]	0.019 *
**Erythrocyte sedimentation rate, mm/h, M (SD)**	36.0 [26.0; 49.0]	37.7 (14.9)	33.0 (16.9)	0.009 *
**In-hospital mortality, n (%)**	55 (15.3%)	7 (3.1%)	48 (36.4%)	<0.001 *
**Status at 30 days** **, n (%)**	60 (16.7%)	11 (4.8%)	49 (37.1%)	<0.001 *
**History of cerebrovascular disease, n (%)**	145 (40.3%)	83 (36.4%)	62 (47.0%)	0.049 *

Note: For all other clinical, laboratory and instrumental parameters analysed in our study, no statistically significant differences were found (*p* ≥ 0.05 for all). *—statistically significant differences (*p* < 0.05). GFR by CKD-EPI—glomerular filtration rate by Chronic Kidney Disease Epidemiology Collaboration formula; bold font in the table footer is used for better visual guidance.

**Table 4:** Comparison of covariates between groups after propensity score matching.

Variable	Patients not Undergoing PCI (n = 70)	Patients Undergoing PCI (n = 70)	SMD
**Age, years**	81.0 ± 4.7	80.7 ± 4.9	0.05
**ST-segment changes on admission, n (%)**	18 (25.7%)	17 (24.3%)	0.05
**Troponin on admission**	2392.9 ± 7023.1	2672.8 ± 7238.3	0.04
**CK max., U/L**	503.6 ± 1617.5	520.9 ± 742.3	0.01
**Glucose max., mmol/L**	8.2 ± 3.3	8.4 ± 3.1	0.06
**Leukocytes max., ×10^9^/L**	9.8 ± 3.8	9.4 ± 3.3	0.10
**LVEF on day 1, %**	50.6 ± 11.5	51.6 ± 8.2	0.09
**IRCAO, n (%)**	5 (7.1%)	4 (5.7%)	0.04

SMD—standardized mean difference. SMD values < 0.1 indicate successful group balancing. CK—creatine phosphokinase; LVEF—left ventricular ejection fraction; IRCAO—infarct-related coronary artery occlusion; bold font in the table footer is used for better visual guidance.

**Table 5:** Frequency of PCI and 30-day outcomes depending on GRACE risk score.

Parameters	Categories	GRACE Risk Score			*p*
		Low	Moderate	High	
**PCI performed, n (%)**	Yes	6 (60.0%)	24 (51.1%)	248 (81.8%)	<0.001 * p_high–moderate_ < 0.001
**Status at 30 days, n (%)**	Deceased	0 (0.0%)	2 (4.3%)	58 (19.1%)	0.014 * p_high–moderate_ = 0.035

Note: PCI—percutaneous coronary intervention; GRACE—Global Registry of Acute Coronary Events. *—statistically significant differences (*p* < 0.05).

## Data Availability

The datasets presented in this article are not readily available because the data are part of an ongoing study.

## Abbreviations

The following abbreviations are used in this manuscript:

ACS	Acute coronary syndrome
AMI	Acute myocardial infarction
CA	Coronary artery
CAD	Coronary artery disease
CABG	Coronary artery bypass grafting
CHF	Chronic heart failure
CI	Confidence interval
CK	Creatine phosphokinase
ECG	Electrocardiogram
GRACE	Global Registry of Acute Coronary Events
HR	Hazard ratio
IQR	Interquartile range
IRCAO	Infarct-related coronary artery occlusion
LVEF	Left ventricular ejection fraction
M	Mean
Me	Median
MI	Myocardial infarction
NSTEMI	Non-ST-segment elevation myocardial infarction
NSTE-ACS	Non-ST-segment elevation acute coronary syndrome
PCI	Percutaneous coronary intervention
PSM	Propensity score matching
SD	Standard deviation
SMD	Standardized mean difference
STEMI	ST-segment elevation myocardial infarction
SYNTAX	Synergy between PCI with TAXus and Cardiac Surgery
TIMI	Thrombolysis in Myocardial Infarction
